# Sphenoid sinus mucocele: an unusual complication of head and neck irradiation in a North African woman

**DOI:** 10.4317/jced.55161

**Published:** 2019-02-01

**Authors:** Aina Brunet-Garcia, Mª Dolores Costa-Climent,, Maria Pujol-Rodríguez, Laia Brunet-Garcia, Marta Faubel-Serra

**Affiliations:** 1 MD, Department of Otorhinolaryngology, Hospital Universitar Parc Taulí. Sabadell, Barcelona. Universitat Autònoma de Barcelona. Universitat de València. Spain; 2 MD, Department of Otorhinolaryngology, Hospital General Universitari de Castelló. Castelló de la Plana, Castelló, Spain; 3 MD, Department of Otorhinolaryngology, Hospital Universitari Vall d’Hebron. Barcelona. Spain; 4 MD, Department of Paediatric Cardiology, Hospital de Mataró. Department of Pediatrics, Hospital Sant Joan de Déu, Barcelona. Universitat de Barcelona. Spain; 1 MD, PhD. Head of Otorhinolaryngology department, Hospital General Universitari de Castelló. Castelló de la Plana, Castelló, Spain

## Abstract

Mucocele is a common benign lesion otherwise rarely located in the sphenoid sinus. Some complications after head and neck irradiation have been described in the literature until now. To our knowledge, this is the first report of a sphenoid sinus mucocele in a North African patient treated some years before with radiotherapy for a nasopharyngeal carcinoma (NPC). We extend the literature review about this infrequent finding, of which head and neck surgeons should be aware.

** Key words:**Mucocele, sphenoid sinus mucocele, nasopharyngeal carcinoma, radiotherapy, North African.

## Introduction

Isolated sphenoid sinus mucocele is a rare entity. Its incidence has been reported to be 1-3% of all mucoceles.

Diagnosis of sphenoid sinus mucocele is often delayed due to its late symptoms that are not evident until it expands and compresses neighbouring structures. Furthermore, this location makes it impossible to direct examination (1). Correlation between radiotherapy and mucocele formation has rarely been described in the literature. We report a case of a North African woman that developed sphenoid sinus mucocele 11 years after receiving radiotherapy for a nasopharyngeal carcinoma.

## Case Report

A 49 year-old female patient originally from Maghreb was referred to our Department due to some abnormalities found on a magnetic resonance imaging (MRI) developed for temporomandibular joint dysfunction.

The patient had undergone radiotherapy for a NPC 11 years before.

**Figure 1:**
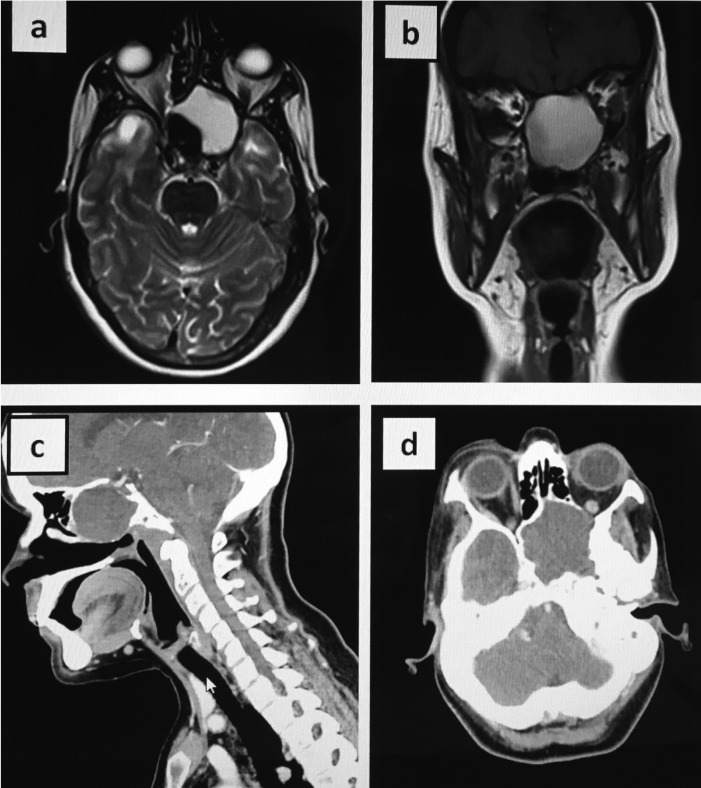
Radiological Images: Magnetic Ressonance (MR). Mucocele shows high signal intensity in both T1 and T2 weighted images. a: T2 axial MR, b: T1 coronal MR, c: sagital CT, d: axial CT.

MRI revealed a cystic mass located in the clivus area (Fig. 1a,b). Contrast computed tomography scan (CT) showed a well-defined mass with low attenuation and unenhanced by contrast agents, located in the sphenoid bone (Fig. 1c,d). Nasal endoscopic examination showed a cystic mass in the upper side of the cavum.

The patient had gone to the Emergency Unit complaining about visual disturbances some weeks before.

**Figure 2:**
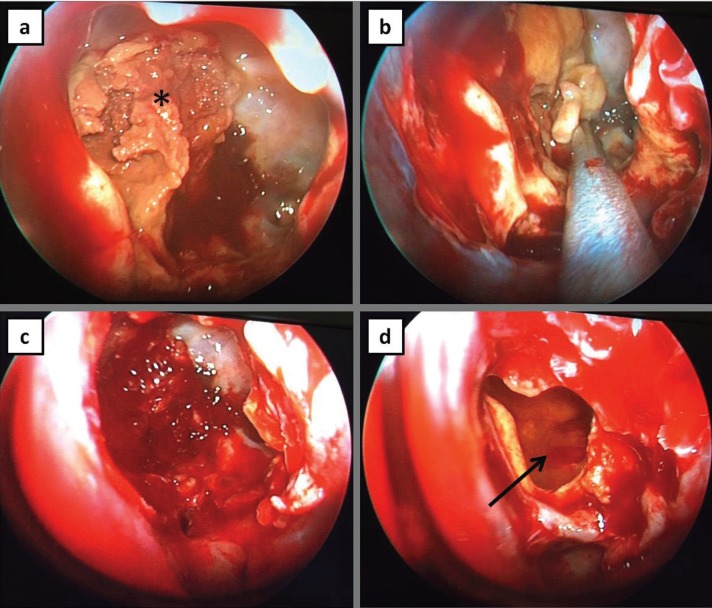
Surgical images. a: detritus material inside sphenoid sinus (star), b: suction of the mucocele, c,d: empty sphenoid sinus cavity after mucocele excision (arrow).

Under general anaesthesia marsupialisation of the mucocele was performed, and a thick yellowish mucopurulent fluid was aspired from the mass (Fig. 2).

**Figure 3:**
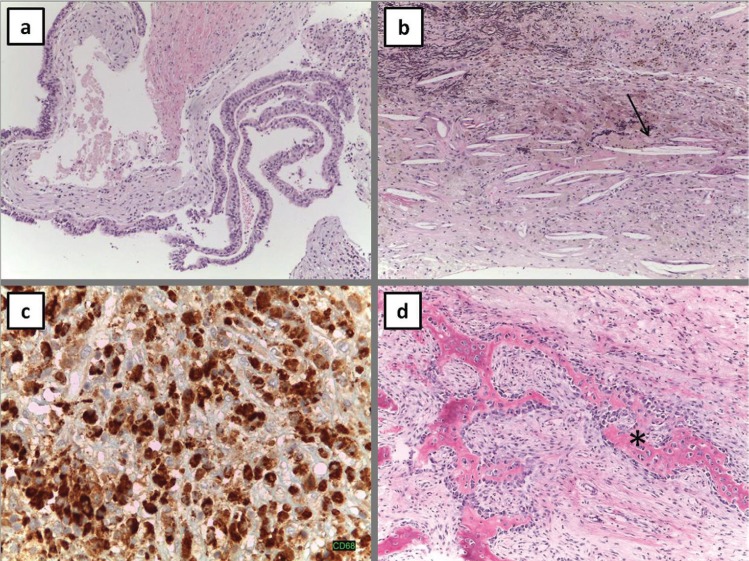
Histopathological examination images. a: Cyst lined by respiratory epithelium, b: Inflammatory cells and cholesterol crystals (arrow), c: inflammatory cells, d: hemorrage areas.

Pathological findings of the lining mucosa showed a mucous retention cyst with no evidence of tumor cells (Fig. 3). Intraoperative cultures were sterile. Visual symptoms disappeared after surgery. The patient had regular follow-ups with no evidence of recurrence during 4 years.

Informed consent was obtained by the patient.

## Discussion

Mucoceles are considered the most frequent expansive lesions of the paranasal sinuses (1). They are most commonly located in the frontal sinus (60-65%), followed by the ethmoid (20-30%), maxilar (10%) and sphenoid sinus (1-3%) (2).

Mucoceles are considered to arise due to sinus ostium obstruction, preceded by fibrosis, infection, trauma, surgery (35-66%), fibrous dysplasia, osteoma or ossifying fibroma. Radiotherapy for a NPC is a rare ethiology for this entity; scarcely any case has been described in the literature before (2).

Although being rare in Caucasian, it is endemic in southern China. Southeast Asia, North Africa, the Middle East and the Arctic are considered intermediate-risk regions (3).

Nasal endoscopy followed by a CT scan or an MRI are useful tools for the diagnosis, since physical examination might be normal. CT scan would be characterized by a sinus homogeneous isodense mass which does not enhance with contrast administration; it totally fills the sinus cavity and surrounding structures. Mucoceles usually show moderate or low signal intensity on T1 weighted MRI images and high signal intensity on T2 weighted images, although in some cases there is a high protein content and density, which leads to T1 high intensity images, as in the present case (4).

Porter *et al.* analysed paranasal sinuses CT scans before and after radiotherapy in patients with NPC. They could find an increase in chronic sinus disease due to mucous membrane atrophy, as a late complication of radiotherapy (4). The most common side effects of radiation therapy for a NPC are xerostomia (98%), eustachian tube fibrosis causing serous otitis media, fibrosis of the temporomandibular joint leading to the appearance of trismus, and neck fibrosis causing neck stiffness, with an incidence from 40 to 70% (2).

Local recurrence was improbable in this case due to CT and MRI characteristics, endoscopic examination findings, the presence of a previous nasosinusal surgery, and long time (11 years) after radiotherapy, since only a 9% of recurrence arising 5 years after radiotherapy has been reported (5).

Endoscopic drainage, which is effective and secure, is considered the outstanding treatment for this entity (6).

Only few cases of sphenoid mucocele after irradiation for NPC have been reported in the literature, most of them being chinese male patients ( Table 1). Common presentation signs were headache (7,8) and visual disturbances (1,8-11). To our knowledge this complication of radiotherapy has never been mentioned before in a North African patient. The role of radiotherapy as a primary cause of this entity was first described by Rejab *et al.* in 1991. In that case authors supported the idea that sinus ostium was occluded by scarred mucosa following radiotherapy, leading to mucocele formation (9), similarly to the present case.

**Table 1:**
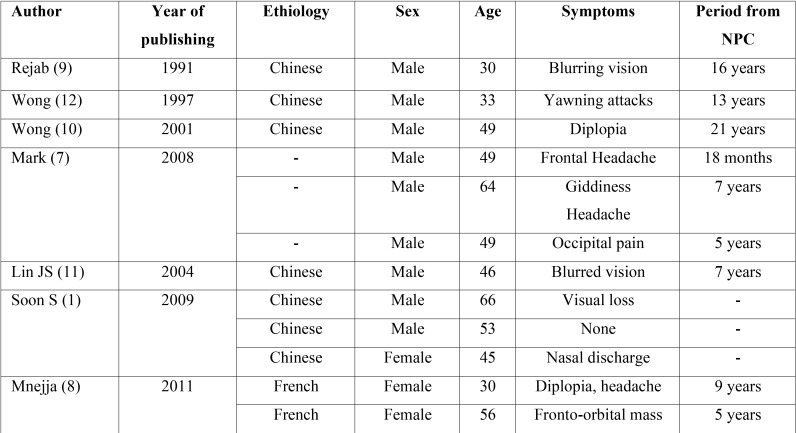
Patients that developed sphenoid sinus mucocele after radiotherapy for nasopharyneal carcinoma (NPC).

## Conclusions

Sphenoid sinus mucocele developing after radiotherapy for NPC has rarely been reported and it has been mostly described in chinese man. Its presentation may sometimes mimic recurrence and hence, clinical history, examination, CT scanning and MRI are useful tools for the diagnosis. It is crucial for oral surgeons and otorhinolaryngologists to keep this uncommon entity in mind in order to allow early diagnosis and treatment and prevent possible complications developing in the orbits or intracranially.
